# Encephalomyocarditis Virus 2A Protein Inhibited Apoptosis by Interaction with Annexin A2 through JNK/c-Jun Pathway

**DOI:** 10.3390/v14020359

**Published:** 2022-02-09

**Authors:** Ruochan Han, Lin Liang, Tong Qin, Sa Xiao, Ruiying Liang

**Affiliations:** 1 Institute of Animal Sciences, Chinese Academy of Agricultural Sciences, Beijing 100193, China; hanruochan@126.com (R.H.); lianglin@caas.cn (L.L.); qintong@caas.cn (T.Q.); 2 College of Veterinary Medicine, Northwest A&F University, Yangling 712100, China

**Keywords:** encephalomyocarditis virus, 2A, apoptosis, annexin A2, JNK/c-Jun pathway

## Abstract

Encephalomyocarditis virus can cause myocarditis and encephalitis in pigs and other mammals, thus posing a potential threat to public health safety. The 2A protein is an important virulence factor of EMCV. Previous studies have shown that the 2A protein may be related to the inhibition of apoptosis by virus, but its specific molecular mechanism is not clear. In this study, the 2A protein was expressed in *Escherichia coli* in order to find interacting cell proteins. A pull down assay, coupled with mass spectrometry, revealed that the 2A protein possibly interacted with annexin A2. Co-immunoprecipitation assays and confocal imaging analysis further demonstrated that the 2A protein interacted with annexin A2 in cells. In reducing the expression of annexin A2 by siRNA, the ability of the 2A protein to inhibit apoptosis was weakened and the proliferation of EMCV was slowed down. These results suggest that annexin A2 is closely related to the inhibition of apoptosis by 2A. Furthermore, both RT-PCR and western blot results showed that the 2A protein requires annexin A2 interaction to inhibit apoptosis via JNK/c-Jun pathway. Taken together, our data indicate that the 2A protein inhibits apoptosis by interacting with annexin A2 via the JNK/c-Jun pathway. These findings provide insight into the molecular pathogenesis underlying EMCV infection.

## 1. Introduction

Encephalomyocarditis virus (EMCV) is a small non-enveloped single-stranded RNA virus, which can cause myocarditis, encephalitis, neurological diseases, reproductive disorders, and diabetes in several mammalian species [1]. The genome of EMCV is approximately 7.8 kb and encodes a polyprotein. The EMCV 2A protein is a small protein of approximately 17 kDa and is considered an important virulence protein [1,2]. Previous studies have shown that the 2A protein plays an important role in inhibiting protein synthesis and apoptosis in host cells [1]. It was reported that BHK21 cells infected by EMCV with the deletion of 2A causes apoptosis through caspase 3 activation [3], and it was suggested that the 2A protein is required for the inhibition of apoptosis. However, the specific molecular mechanisms by which the 2A protein inhibits apoptosis remain unclear. In this study, we demonstrated that the EMCV 2A protein inhibits apoptosis, and annexin A2 of the host cells plays an important role in the mechanism of 2A protein inhibiting apoptosis.

Annexin A2 is a 36 kDa protein and contains three distinct functional regions, including the N terminus, the core domain, and the C terminus [4]. Annexin A2 exists in cells in two forms. One is a monomer that mainly exists in the cytoplasm [5] and participates in the assembly, dissolution, and repair of intracellular organelle membranes [6]. The other is a heterotetramer which mainly exists on the surface of cell membranes, and is formed by two molecules of annexin A2 and two molecules of P11 [5]. Annexin A2 has been implicated in multiple diseases, immune function, and viral infection. Previous studies have shown that it can regulate cell cycles by inhibiting apoptosis. In non-small-cell lung cancer (NSCLC) cells, annexin A2 activated JNK/c-Jun signaling, which, in turn, led to a decrease in p53 transcription [7]. Our research demonstrates that the 2A protein inhibits apoptosis by interacting with annexin A2 via the activation of the JNK/c-Jun pathway during the early stage of EMCV replication in BHK21 cells.

## 2. Methods

### 2.1. Cells, Plasmids and Viruses

PK15 and BHK21 cells were cultured in Dulbecco’s modified Eagle’s medium (DMEM) (Invitrogen, Carlsbad, CA, USA), supplemented with 5% and 8% heat-inactivated fetal bovine serum (FBS) (Gibco, California USA) respectively, at 37 °C in a 5 *v*/*v* CO2 humidified atmosphere. The EMCV-HB10 strain (GenBank accession number: JQ864080.1) and pcDNA3.1-2A plasmid were kept in laboratory.

### 2.2. Prokaryotic Expression of 2A Protein

Using the pcDN3.1-2A plasmid as a template, the 2A gene was amplified using the PCR primer pair His-2A (Table 1). Recombinant plasmid His-2A was constructed by cloning the 2A gene into the pET-28a-sumo vector. The His-2A plasmid was transformed into *E. coli* BL21 (DE3) cells to obtain the recombinant fusion protein His-2A. The His-2A protein was purified using His-tag protein purification beads. The purified recombinant fusion protein was analyzed by sodium dodecyl sulfate-polyacrylamide gel electrophoresis (SDS-PAGE).

### 2.3. His Pull Down Assay

PK15 cells cultured in flasks were washed twice with PBS. A lysis buffer (Thermo Fisher Scientific, Waltham, MA, USA) and protease inhibitor were added. After incubating the cells at 4 °C for 30 min, the lysed cells were centrifuged at 3000× *g* for 15 min at 4 °C. The supernatant was harvested for His pull-down assay. The purified His-2A protein was incubated with the supernatant at 4 °C overnight. After incubation, the mixture was conjugated to His-tag beads for 1.5 h at room temperature. The beads were washed twice with washing buffer. Then, the His-2A protein and interacting proteins were eluted with elution buffer containing 20 mM phosphate buffer, 500 mM NaCl, and 500 mM imidazole, followed by the detection of the proteins by SDS-PAGE.

### 2.4. Mass Spectrometry

Binding specificity to the 2A protein by SDS-PAGE was analyzed by mass spectrometry. Protein slices in fresh CCB-stained gel were excised, destained twice with 50 *v*/*v* acetonitrile and 50 mM NH4HCO3, and dried with acetonitrile three times. Then, the dried gel slices were incubated in ice-cold digestion solution (20 mM NH4HCO3 and trypsin 12.5 ng μL^−1^) at 37 °C overnight. Finally, peptides in the supernatant were collected after extraction twice with extract solution (5 *v*/*v* formic acid in 50 *v*/*v* acetonitrile). The peptides collected in the previous step were analyzed by Nano-HPLC-MS/MS. Tandem mass spectra were extracted by Proteome Discoverer software (version 2.4; Thermo Fisher Scientific, Waltham, MA, USA). Peptide confidence was set to high, and peptide ion score was set to >20.

### 2.5. Localization of 2A

The eukaryotic expression plasmid, pEGFP-2A, was transfected into PK15 cells with lipofectamine 2000 (Invitrogen, Carlsbad, CA, USA) and cultured at 37 °C for 48 h. The cell membranes were labeled with DiI stain (1,1’-dioctadecyl-3,3,3’,3’-tetramethylindocarbocyanine perchlorate) (C1036; Beyotime, Shanghai, China), and the cell nuclei were counterstained with DAPI (4’,6-diamidino-2-phenylindole) (F6057; Sigma-Aldrich, St. Louis, MO, USA).

### 2.6. Flow Cytometry

The eukaryotic expression plasmid, pcDNA3.1-2A, was transfected into PK15 cells treated with kevetrin hydrochloride (KH, product number T3184; TargetMol, Shanghai, China). KH-treated and untreated cells were used as positive and negative controls, respectively. Apoptosis of cells was detected using an ANNEXINV-FITC/PI apoptosis detection kit (CA1020; Solarbio, Beijing, China). First, cells were washed with cold PBS and resuspended in 1× binding buffer. Next, annexin V-FITC was added into the suspension at room temperature for 10 min in the dark. Then, PI was added by incubation for 5 min. Finally, cell apoptosis was evaluated using the FACSVerse flow cytometer. The annexin V+ plus annexin V+ PI+ population was gated for apoptosis analysis, and at least 1 × 10^4^ cells event^−1^ were evaluated for each analysis.

### 2.7. Caspase 3 Activity Detection

The eukaryotic expression plasmid, pcDNA3.1-2A, was transfected into PK15 cells. Apoptosis of cells was detected using a Caspase 3 Activity Assay kit (C1115; Beyotime, Shanghai, China). Cells were lysed with lysis buffer at 0 °C for 15 min. And then the lysates were centrifuged at 15,000× *g* for 15 min at 4 °C. The protein concentrations in the supernatant were determined by BCA protein assay (Thermo Fisher Scientific, Waltham, MA, USA). Protein extracts (30 μg) were incubated in a 96-well microtitre plate with 20 ng Ac-DEVD-pNA for 2 h at 37 °C. Caspase 3 activity was measured by cleavage of the Ac-DEVD-pNA subsatrate to pNA, the absorbance of which was measured at 405 nm. Relative caspase activity was calculated as the ratio of emission of treated cells to untreated cells.

### 2.8. Confocal Imaging and Co-IP

The pDsRed-ANXA2 and HA-ANXA2 plasmids encoding annexin A2 (GenBank accession number XM_013992912.2) and the pEGFP-2A and Flag-2A plasmids were constructed (Table 1). To further identify interactions involving 2A and annexin A2 proteins, the appropriate plasmids were transfected into PK15 cells using Lipofectamine 2000. After 48 h, cells analyzed for confocal microscopy and cell nuclei were labeled with DAPI.

PK15 cells were transfected with the HA-ANXA2 and Flag-2A plasmids. After washing with cold PBS, cells were lysed with IP lysis buffer (26149; Thermo Pierce, Shanghai, China), PMSF, and protease inhibitor cocktail (26149; Thermo Pierce, Shanghai, China) at 4 °C for 1 h. The obtained cellular proteins were pre-cleared with protein A/G beads (26149; Thermo Pierce, Shanghai, China) and incubated with protein A/G beads plus and rabbit anti-HA-specific (C29F4; Cell Signaling Technology, Boston, USA) antibody at 4 °C overnight. Then, the beads were washed with elution buffer and the eluted proteins were analyzed by SDS-PAGE. Finally, immunoblotting analysis of the interacting proteins was performed with mouse anti-Flag (F1804; Sigma-aldrich, St. Louis, MO, USA) pcAb and HRP-conjugated goat anti-mouse secondary antibodies.

### 2.9. siRNA Interference

siRNAs specific for annexin A2 were designed and synthesized by Sangon Biotech (Table 2) (employing the Rosetta algorithm in their pipeline to design siRNAs; National Center for Biotechnology Information (NCBI) BLAST was used for off-target analysis). The final concentration of the siRNAs used were 100 pM. A non-targeting siRNA was used as the negative control. The siRNAs were transfected into cells by Lipofectamine 2000. The cells (5 × 10^6^ cells mL^−1^) were treated with siRNA1 for 24 h, and pcDNA3.1-2A at different concentrations were transfected in the cells; the results were observed after 24 h. Kevetrin hydrochloride-treated (10 mM) cells were used as a positive control.

### 2.10. Replication Kinetics of EMCV

The titers of EMCV HB10 were determined by endpoint dilution assays, and the 50% cell culture infection dose (TCID_50_) was calculated to determine the replication kinetics of the virus in BHK21 cells. Briefly, cells were seeded onto 96-well microtiter plates and infected with 10-fold serial dilutions of EMCV (1:10^−1^ to 1:10^−8^) separately after siRNA treatment for 24 h. The cytopathic effect (CPE) of the cultures was observed at different time intervals up to 48 h post infection (hp.i.). Assays were performed in triplicate. Each data point represents the average of three independent experiments.

### 2.11. RNA Extraction and Quantitative Real-Time PCR

The mRNAs of cells were obtained by the Cell RNA Rapid Extraction Kit (Aidlab, Beijing, China). Fastking one-step genomic cDNA first strand synthesis Kit (TIANGEN, Beijing, China) was used for reverse transcription. Quantitative real-time PCR assay (RT-PCR) was performed using Universal SYBR^®^ qPCR (Vazyme, Nanjing, China) on the CFX96 Real-time PCR Detection System (Bio-Rad, Hercules, CA, USA). The relative expression levels of mRNAs were analyzed using the 2^−^^ΔΔCt^ method. Glyceraldehyde-3-phosphate dehydrogenase (GAPDH) mRNA was used as an internal standard for quantitative analysis of mRNAs. The primers used in the RT-PCR assay are listed in Table 3, and all reactions were performed in triplicate.

### 2.12. Western Blot

The cells were lysed with lysis buffer (Thermo Fisher Scientific), PMSF, and a protease inhibitor cocktail (Thermo Fisher Scientific) at 4 °C for 30 min. Then, the lysates were centrifuged at 3000× *g* for 15 min at 4 °C. The protein concentrations in the supernatant were determined by BCA protein assay (Thermo Fisher Scientific). Protein extracts (40 μg) were detected by SDS-PAGE and western blot. Polyvinylidene fluoride membranes (PVDF) (Millipore A) were blocked with 5% nonfat milk in TBST at room temperature for 1 h. In turn, the membranes were immunoblotted with primary antibodies at room temperature for 2 h and incubated with secondary horseradish peroxidase-conjugated antibody at room temperature for 1 h. Finally, protein bands were detected by the enhanced chemiluminescence western blot detection kit (Solarbio, Beijing, China). The monoclonal rabbit anti-mouse caspase-3, caspase-8, caspase-9, p-c-Jun (Affinity), and p53, JNK (phospho-Thr183/Tyr185) Abs (Sangon Biotech, Shanghai, China) and the monoclonal goat anti-mouse β-actin Abs (Beyotime, Shanghai, China) were used.

### 2.13. Statistical Analyses

Data are reported as means ± SD of 3 independent experiments and analyzed using GraphPad^TM^ Prism software (version 6; GraphPad Software, San Diego, CA, USA).

## 3. Results

### 3.1. 2A Protein Inhibits Apoptosis in PK15 Cells

**Figure 1:**
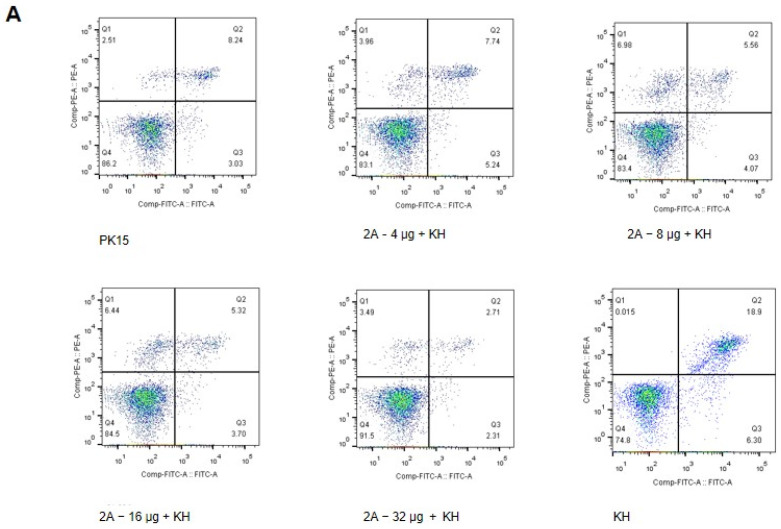

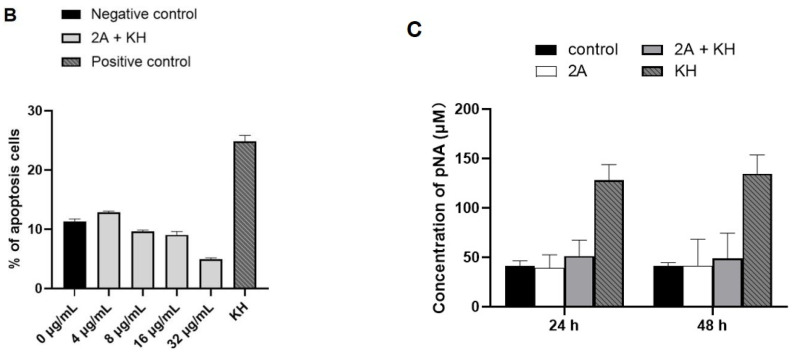
2A protein inhibits apoptosis in PK15 cells. (**A**) PK15 cells were treated with KH and different concentrations of pcDNA3.1-2A for 24 h and analysed by flow cytometry. (**B**) Percentages of Annexin-V-FITC and PI-positive cells from gated cells. (**C**) The changes of caspase 3 in PK15 cells induced by 8 μg mL^−1^ pcDNA3.1-2A for 24 h or 48 h. The data are presented as the mean values and standard deviations from three independent experiments.

Kevetrin hydrochloride (KH) is an activator of the protein p53 and induces apoptosis. The cells treated with KH were used as a positive control. KH-treated cells were treated with or without pcDNA3.1-2A for 24 h to determine whether the 2A protein inhibited the apoptosis of PK15 cells, and they were then stained with annexin V and propidium iodide (PI) to detect the rate of apoptosis by flow cytometry (Figure 1A). The results revealed significant differences in the proportions of apoptotic cells; 24.8 *v*/*v* ± 0.1 *v*/*v* of the cells were apoptotic in the KH-treated group, whereas 12.88 *v*/*v* ± 0.1 *v*/*v* were apoptotic in the groups treated with 4 µg mL^−1^ pcDNA3.1-2A. These results demonstrated that the 2A protein can inhibit KH induced apoptosis, and has a dose-dependent apoptosis inhibiting effect upon treatment with 4 to 32 µg mL^−1^ pcDNA3.1-2A

Meanwhile, we used a caspase 3 activity assay kit to detect the cleavage of caspase 3 in PK15 cells treated with 8 μg mL^−1^ pcDNA3.1-2A for 24 h and 48 h. Compared with the results of KH control group, there was no significant difference in caspase 3 of 2A infected cells. The results showed that pcDNA3.1-2A-treated cells inhibited the activation of caspase 3 compared to that in untreated control cells (Figure 1C). These results demonstrated that the 2A protein can inhibit apoptosis.

### 3.2. 2A-Interacting Proteins Identified by His Pull-Down Assay and Mass Spectrometry

**Figure 2:**
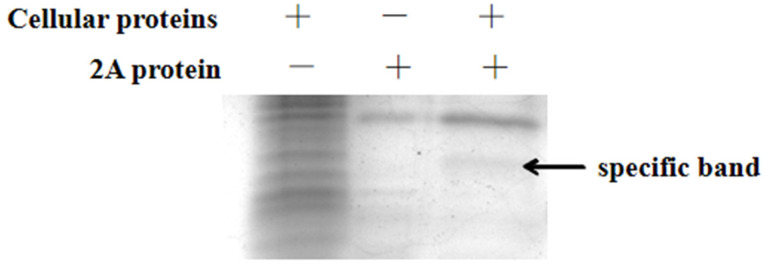
Identification of 2A-interacting protein by His pull-down assay. Lane 1, proteins of PK15 cells; lane 2, purified 2A protein; lane 3, the specific proteins that may interact with 2A protein by pull down assay. 2A protein produced in *E. coli* BL21 (DE3) was used for the His pull-down assay.

In order to explore the molecular mechanism of 2A protein regulating apoptosis, we found the cell proteins that may interact with the 2A protein by pull-down assay. To identify cellular proteins that potentially interact with the 2A protein, the His pull-down assay was coupled with mass spectrometry. The specific protein band in the His-2A lane (Figure 2) was excised and subjected to mass spectrometric analysis. Cellular proteins that potentially interact with the 2A protein were identified in this analysis (Table 4). Based on the previous literatures, annexin A2 is closely related to viral pathogenicity and apoptosis [8,9,10]. We chose annexin A2 as the interaction protein with the 2A protein for further study.

### 3.3. 2A Protein Interacts and Colocalizes with Annexin A2

**Figure 3:**
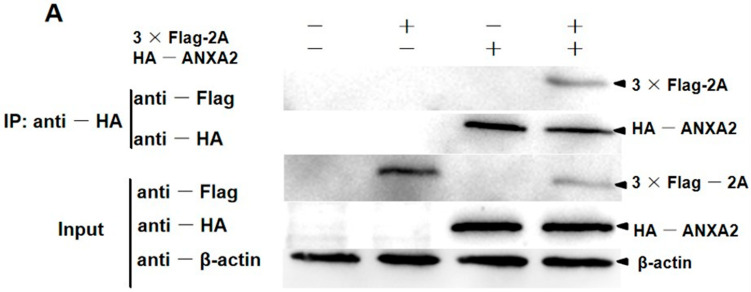

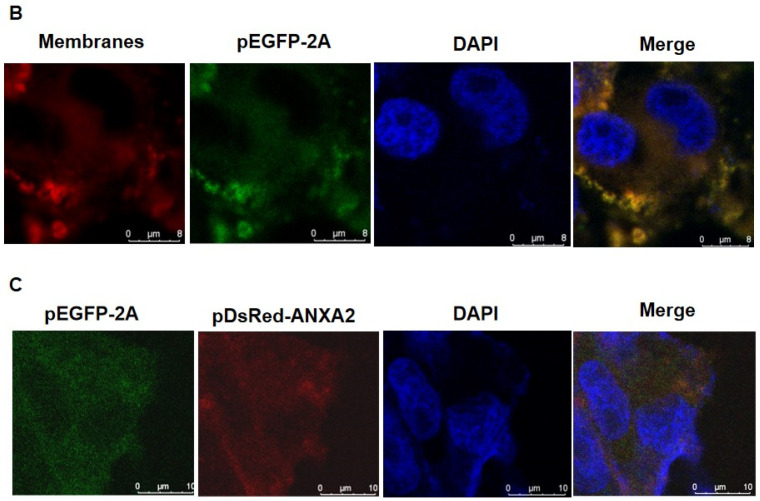
Identification of the interaction between the 2A protein and annexin A2. (**A**) The results of Co-IP. The plasmid (+) or empty vectors (−) were transfected into PK15 cells, and the whole-cell lysates obtained at 48 h post-transfection were immunoprecipitated (IP) with anti-HA pcAb. Then, proteins separated by SDS-PAGE were detected by immunoblotting with the indicated antibodies. The proteins were labeled on the right of the band. (**B**) Colocalization of the 2A protein with endogenous annexin A2 which is in the membranes. The PK15 cells were stimulated with 4 μg mL^−1^ pEGFP-2A. Cells were fixed 48 h after plasmids transfection and examined by confocal microscopy to detect 2A (green) and membranes (red). The membranes were labeled with DiI (red) stain. The position of the nucleus is indicated by DAPI (blue) staining in the merged image. Bars = 8 μm. (**C**) Colocalization of 2A protein and annexin A2. PK15 cells were co-transfected with pEGFP-2A and pDsRed-ANXA2. Cells were fixed at 48 hpt and examined by confocal microscopy to detect 2A (green) and annexin A2 (red). The position of the nucleus is indicated by DAPI (blue) staining in the merged image. Bars = 10 μm. Experiments were performed at least three times.

To further confirm the interaction between the 2A protein and annexin A2, Flag-2A and HA-ANXA2 alone or both were transiently expressed in PK15 cells. Cells co-expressing 3 × Flag and the HA-ANXA2 protein were used as a negative control. Co-immunoprecipitation (Co-IP) with an anti-HA monoclonal antibody (MAb) showed that the HA-ANXA2 protein interacted with 3 × Flag-2A but not with 3 × Flag (Figure 3A).

pEGFP-2A and pDsRed-ANXA2 alone, or in combination, were transiently expressed in PK15 cells. The subcellular localizations of interactions involving the 2A protein and endogenous annexin A2 on cell membranes and the 2A protein and exogenous annexin A2 overexpressed in PK15 cells, were also shown by confocal microscopy. The interaction between the 2A protein and annexin A2 (a membrane protein) was confirmed by both endogenous and exogenous methods. By transfecting pEGFP-2A alone, the 2A protein was localized on the cytomembrane (Figure 3B). The results of co-transfection pEGFP-2A and pDsRed-ANXA2 showed that both the 2A protein and annexin A2 were distributed throughout the cytoplasm, and annexin A2 colocalized extensively with 2A protein (Figure 3C).

### 3.4. Reducing Annexin A2 Using Small Interfering RNA (siRNA) Induces Apoptosis

**Figure 4:**
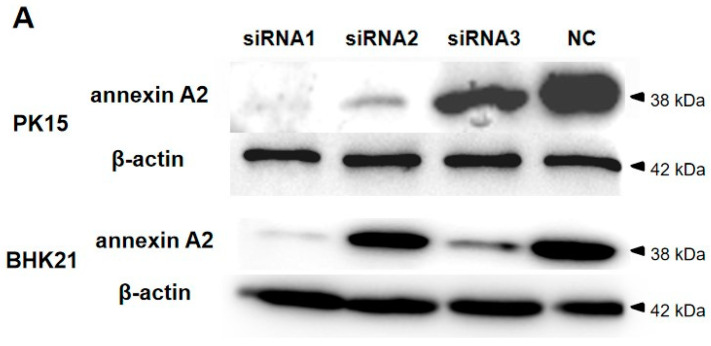

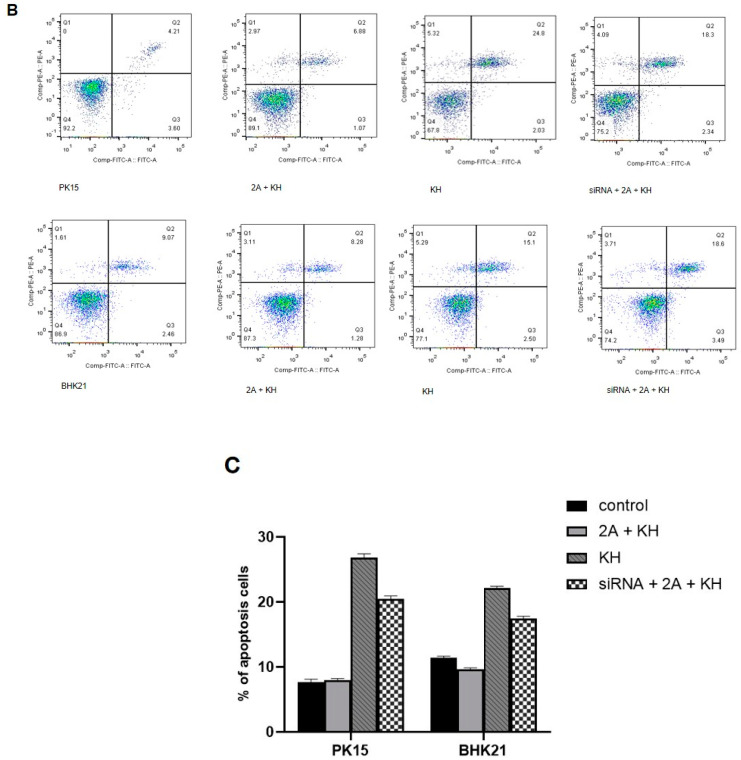
Reducing of annexin A2 attenuated the apoptosis inhibited by the 2A protein. (**A**) Reducing of annexin A2 expression by siRNA treatment. PK15 and BHK21 cells transfected with negative siRNA (NC) or treated with siRNAs separately against annexin A2 (100 pM siRNA1, siRNA2, or siRNA3) were harvested at 48 hpt. Endogenous annexin A2 of cells was detected by immunoblotting with anti-annexin A2 Abs. (**B**) The apoptosis levels of PK15 and BHK21 cells were detected by flow cytometry. Cells treated with 100 pM siRNA1 for 24 h were transfected with the indicated concentrations of pcDNA3.1-2A and added KH for 24 hpt. (**C**) Percentages of Annexin-V-FITC and PI positive cells from gated cells. The data are presented as the mean values and standard deviations from three independent experiments.

siRNA interference was used to investigate whether the 2A protein inhibits apoptosis by interacting with endogenous annexin A2 in cells. Endogenous annexin A2 expression in PK15 and BHK21 cells was reduced by siRNAs targeting annexin A2 respectively (Figure 4A). The levels of PK15 and BHK21 cells apoptosis inhibited by the 2A protein were evaluated by flow cytometry (Figure 4B,C). Cells treated with 10 mM KH were used as a positive control, separately. When PK15 and BHK21 cells overexpressed the 2A protein and were treated with KH, apoptosis decreased. This shows that 2A has an inhibitory effect on KH-induced apoptosis. When the 2A protein was overexpressed in PK15 and BHK21 cells with reduced annexin A2 expression and then treated with KH, there was only a small reduction in apoptosis compared with the positive control. The data show that annexin A2 plays an important role in 2A inhibiting apoptosis. In summation, these data suggest that the 2A protein inhibits apoptosis by interacting with annexin A2 in both PK15 and BHK21 cells.

### 3.5. Reducing of Annexin A2 Increased Apoptosis in the Infection of EMCV

**Figure 5:**
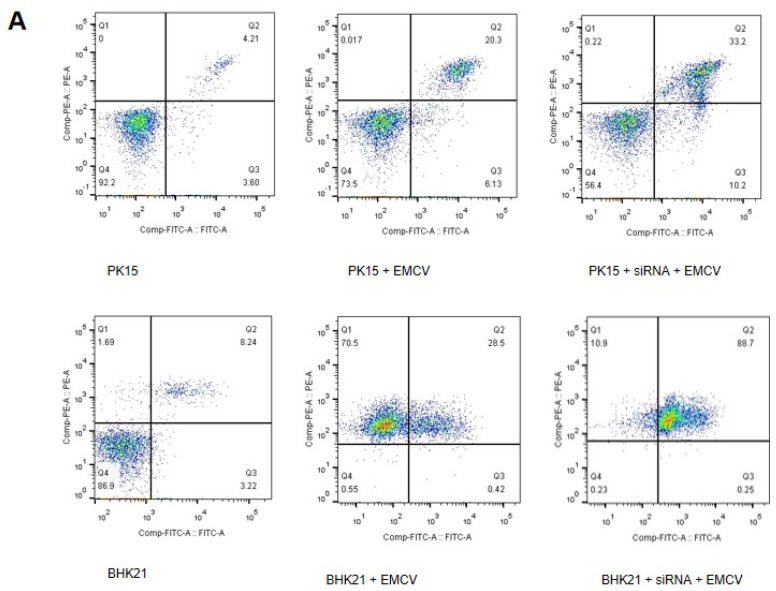

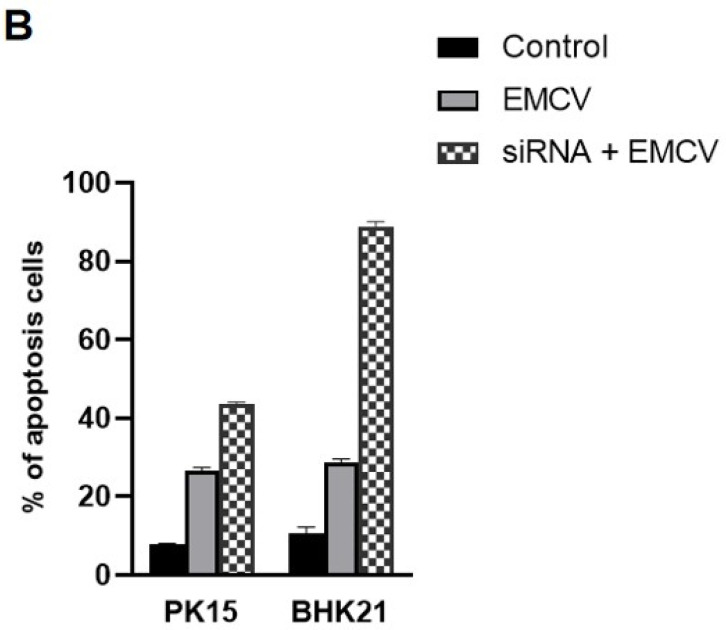
Reducing of annexin A2 increased apoptosis in the infection of EMCV. (**A**) The apoptosis levels of PK15 and BHK21 cells were detected by flow cytometry. PK15 and BHK21 cells treated with 100 pM siRNA1 were infected with MOI of 0.01 with EMCV-HB10 for 24 hpt. (**B**) Percentages of Annexin-V-FITC and PI positive cells from gated cells. The data are presented as the mean values and standard deviations from three independent experiments.

EMCV has been described as a lytic virus in BHK21 cells and promotes virus proliferation by inhibiting apoptosis [1]. Cells of PK15 and BHK21 treated with siRNAs targeting annexin A2 were then infected with EMCV. Reducing annexin A2 increased apoptosis in the infection of EMCV. The levels of cells apoptosis were evaluated by flow cytometry (Figure 5A,B).

### 3.6. Effect of Annxin A2 on EMCV Proliferation

**Figure 6:**
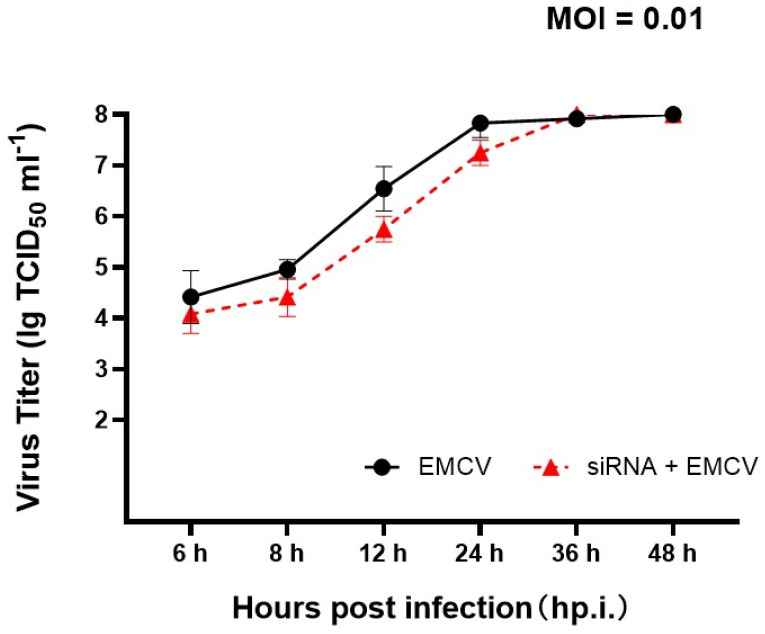
Effect of annexin A2 on EMCV proliferation. One-step growth curves of EMCV-HB10 in BHK21 (●) and BHK21 reducing annexin A2 (▲). An MOI of 0.01 was used for infection, supernatant and cells were harvested at each indicated time and freeze-thawed together for viral titration, as described in Methods.

To characterize the effect of annexin A2 on EMCV proliferation, the growth kinetics of EMCV-HB10 in BHK21 and BHK21 reducing of annexin A2 were compared (Figure 6). The one-step growth curve of EMCV-HB10 in BHK21 cells reducing annexin A2 reached the plateau 12 h later than EMCV-HB10 in BHK21 cells. The two growth curves tended to be consistent after 36 h, which may be related to the effect of siRNA interference.

### 3.7. Annexin A2 Mediates the Inhibition of Apoptosis by 2A Protein via JNK/c-Jun Pathway in BHK21 Cells

**Figure 7:**
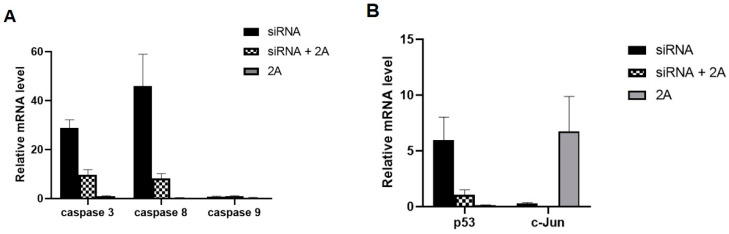

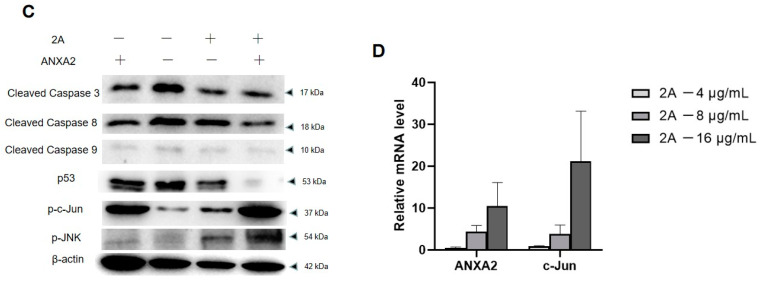

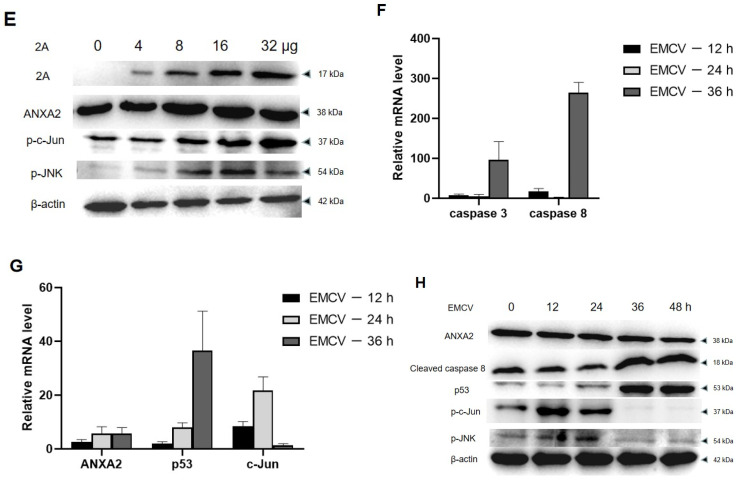
The 2A protein inhibits apoptosis by interacting with annexin A2 via JNK/c-Jun pathway in BHK21 cells. (**A**) BHK21 cells were treated with annexin A2 siRNA1 alone, or together with pcDNA3.1-2A or alone with pcDNA3.1-2A were harvested at 48 hpt. The mRNA expression of caspase 3/8/9 were detected by Real-time RT-PCR analysis. GAPDH was used as an internal control. (**B**) Cells were treated in the same way as (**A**), the mRNA expression of p53 and c-Jun were detected by RT-PCR analysis. (**C**) Cells were treated in the same way as (**A**), the expression of caspase 3/8/9, p53, p-JNK and p-c-Jun were detected by Western blot. Anti-β-actin antibody was used to confirm comparable loading. (**D**) BHK21 cells were treated with different concentrations of pcDNA3.1-2A for 24 h, and then the mRNA expression of annexin A2 and c-Jun were detected by RT-PCR analysis. (**E**) BHK21 cells were treated as (**D**) for 48 h, and then the expression of 2A, annexin A2, p-JNK and p-c-Jun were detected by Western blot. (**F**,**G**) BHK-21 cells were infected at an MOI of 0.01 with EMCV, and at each indicated time, supernatant and cells were harvested. The mRNA expression of caspase 3/8, p53 and c-Jun were detected by RT-PCR analysis. (**H**) Cells were treated in the same way as (**F**), the expression of caspase 8, annexin A2, p53, p-JNK and p-c-Jun were detected by Western blot. The data are presented as the mean values and standard deviations from three independent experiments.

A previous study indicated that annexin A2 suppresses the expression of p53 resulting in the decrease of p53 dependent cell apoptosis, and JNK is activated during this process [7]. In this study, BHK21 cells reducing annexin A2 by siRNA were transfected with pcDNA3.1-2A. The changes of JNK/c-Jun pathway factors in the cells were detected by RT-PCR and Western blot. As shown in Figure 7A, reducing annexin A2 led to the increase of caspase 3/8 activity in BHK21 cells but had no effect on caspase 9. In contrast, increased the 2A protein and reduced annexin A2 significantly decreased the activity of caspase 3/8 in BHK21 cells. BHK21 cells overexpressing the 2A protein did not cause the activation of caspase 3/8/9. At the same time, overexpressed 2A protein also decreased p53 expression and promoted the activation of c-Jun and JNK (Figure 7B,C). Our findings showed that the activation of c-Jun and JNK is directly proportional to the expression of the 2A protein (Figure 7D,E).

The activities of c-Jun and JNK in EMCV-infected BHK-21 cells peaked at 12 and 24 h of infection and decreased at 36 h. Correspondingly, the expression of caspase 3/8 and p53 increased after 36 h of virus infection (Figure 7F,G). At 36 h after EMCV infection, the phosphorylation of c-Jun decreased significantly. The data implicated that other factors are involved in accelerating apoptosis in the late reproductive stage of EMCV. These results showed that the 2A protein inhibits apoptosis by interacting with annexin A2 mainly within 24 h after virus infection. Furthermore, western blot analysis showed the same results (Figure 7H). The changes of these pathway factors indicated that annexin A2 interacted with the 2A protein inhibits apoptosis through JNK/c-Jun pathway in the early stage of virus infection.

## 4. Discussion

EMCV is a small non-enveloped single-stranded RNA virus that belongs to the *Picornaviridae* (pico = small, RNA = ribonucleic acid) family. It can cause myocarditis, encephalitis, and neurological diseases in many mammalian species, and even lead to reproductive disorders and diabetes [1]. The genome of the virus is approximately 7.8 kb, including three unique domains: the VPg protein binding sequence, a poly (c) region, and an internal ribosome entry site (IRES). The open reading frame (ORF) of the EMCV genome encodes a large polyprotein (L-1ABCD-2ABC-3ABCD). The 2A protein is a nonstructural viral protein (approximately 17 kDa and 143 amino acids), and it is an important virulence factor of EMCV [1]. The present study was conducted to enlighten the underlined mechanism associated with EMCV pathogenesis. We specifically targeted the 2A protein, because this protein plays an important role in the pathogenesis of EMCV by different mechanisms. One of most important mechanisms is to break host innate immune response by inhibiting the EMCV infected host cells apoptosis. This protein can also competitively inhibit the synthesis of host protein. Involvement of the 2A protein in host cell apoptosis has already been studied and proven by many researchers [3,11]. However, the underlined, specific mechanism of the 2A protein to inhibit apoptosis is unclear, and it is not related to the ability to shut off codependent translation [3].

Our results indicated that the 2A protein of EMCV strain HB10 inhibits the apoptosis of PK15 cells as indicated by flow cytometry and caspase 3 activity assay kit. Our results are in agreement with Carocci’s [3]. The inhibition of host cell apoptosis by the 2A protein is complemented by annexin A2. Annexin A2, a calcium dependent phospholipid binding protein, is involved in many membrane-related events, such as proliferation, cell-cell adhesion, exocytosis, and endocytosis [12,13,14,15]. Studies have shown that annexin A2 is closely related to the regulation of apoptosis induced by viral infection [16]. Ma et al. confirmed that the NS1 protein of the highly pathogenic H5N1 avian influenza virus interacts with the annexin A2 protein to inhibit apoptosis [8]. Ying et al. showed that the GroEL protein of Mycoplasma gallisepticum induces apoptosis in host cells by interacting with annexin A2 [17]. In our research, results of flow cytometry exhibited that the inhibition of apoptosis by 2A protein was reduced, when annexin A2 expression was inhibited by siRNA. This data indicates that the interaction between annexin A2 and the 2A protein is necessary for apoptosis inhibition by the 2A protein and subsequent EMCV pathogenesis.

By comparing the changes of apoptosis pathway factors under different expression levels of annexin A2 and the 2A protein, the results showed that the 2A protein can promote the increase of c-Jun and inhibit the synthesis of p53. In other words, the 2A protein inhibited apoptosis by interacting with annexin A2 via JNK/c-Jun pathway. During EMCV infecting BHK21 cells, the increase of c-Jun and the degradation of p53 showed that EMCV inhibited apoptosis through JNK/c-Jun pathway in the early stage of infection. It is proved that in the early replication stage of EMCV, the interaction between the 2A protein and annexin A2 inhibits apoptosis to promote virus reproduction. C-Jun N-terminal kinase (JNK) can regulate apoptosis, which is a subfamily of mitogen-activated protein kinases (MAPK). Studies indicate that JNK plays an antiapoptotic role in regulating the survival of cancer cell. The JNK/c-Jun pathway facilitates the invasion of triple negative breast cancer (TNBC) cells [18]. In addition, annexin A2 conducts roles in p53 induced apoptosis in non-small cell lung cancer (NSCLC), it can negatively regulate p53 mRNA expression by activating JNK [7,19]. Our results demonstrate for the first time that annexin A2 is involved in viral regulation of apoptosis through JNK/c-Jun molecular pathway in the early stage of virus replication. The inhibition of apoptosis was eliminated at 36 h of infection. After 24 h, the rapid elimination of c-Jun and the increase of p53 showed that the inhibitory effect of the 2A protein on apoptosis was disappearing, and other factors were involved in accelerating apoptosis.

Furthermore, annexin A2 is closely related to multiple virus replications by affecting attachment, invasiveness, assembly, and release [20,21,22]. The release mechanism of EMCV is not clear. Whether annexin A2 is involved in the release process of newly formed virions needs to be further studied. In addition, whether the decrease in viral titers observed in this study related to the effect of annexin A2 on virus release needs more research to prove. At present, most antiviral drugs are designed to target virus structure and enzymes, which is easy to produce drug resistance. Targeting virus receptors on host cells is an alternative strategy to solve this problem, which can play an antiviral role in the early stage of virus infection. Compounds or short peptides targeting annexin A2 monomers may become the direction of antiviral therapy in the future.

In conclusion, the key finding of our study was the identification of cellular annexin A2 as a novel interaction partner of the 2A protein during inhibition of apoptosis. These results confirmed that the 2A protein could protect EMCV from cellular defense mechanisms by inhibiting cell apoptosis and promoting the spread of virus particles, which is closely related to the pathogenicity of the virus. The mode and molecular mechanisms involving EMCV 2A protein interactions with annexin A2 are still unknown and require further study; exploring these aspects will provide valuable insights for the development of new antiviral target drugs.

## Figures and Tables

**Table 1:** Primers used in the study.

Primer	Sequence (5′–3′) (Restriction Enzyme) ^#^
His-2A-F	cgGGATCCAGTCCAAATGCCCTAGACAT
His-2A-R	cgCTCGAGttaTTGGGTCTGGAAAACCTGTT
Flag-2A-F	cgGTCGACAGTCCAAATGCCCTAGACAT
Flag-2A-R	cgGGATCCttaTTGGGTCTGGAAAACCTGTT
HA-ANXA2-F	cgGGATCCATGTCTACCGTTCATGAAATTCTGTGCAA
HA-ANXA2-R	cgCTCGAGttaTCAGTCATCCCCACCACACAGGTA
pEGFP-2A-F	CGAATTCTGATGAGTCCAAATGCCCTAGACAT
pEGFP-2A-R	GGATCCCGTTGGGTCTGGAAAACCTGTT
pDsRed-ANXA2-F	ctgctcagatctcgagctcaATGTCTACCGTTCATGAAATTCTG
pDsRed-ANXA2-R	tcagttatctagatccggtgTCAGTCATCCCCACCACAC

^#^ Plasmid His-2A, pEGFP-2A, Flag-2A and HA-ANXA2 were constructed by enzyme digestion. The restriction sites are underlined. Plasmid pDsRed-ANXA2 was constructed by Seamless Cloning.

**Table 2:** siRNA used in the study.

siRNA	Sequence (5′–3′)	
	sense	antisense
siRNA1	CCGUCAAAGCAUACACCAATT	UUGGUGUAUGCUUUGACGGTT
siRNA2	GCCUUUGCCUACCAAAGAATT	UUCUUUGGUAGGCAAAGGCTT
siRNA3	CCAAGUGGAUCAGUAUCAUTT	AUGAUACUGAUCCACUUGGTT
NC	UUCUCCGAACGUGUCACGUTT	ACGUGACACGUUCGGAGAATT

**Table 3:** Sequence of oligonucleotide primers used in this study.

siRNA	Sequence (5′–3′)	
	sense	antisense
Caspase 3	AGGTGGGCATCTGGTAGCCA	GATCAGTTATCGCGAATGCCA
Caspase 8	CATCCAGTCACTTTGCCAGA	GCATCTGTTTCCCCATGTTT
Caspase 9	TTCCCAGGTTTTGTTTCCTG	CCTTTCACCGAAACAGCATT
ANXA2	GGTCCAGTGCATTCAGAACA	TCAGTCATCCCCACCACACA
c-Jun	AGAATACGCTGCCCAGTGTC	TAGACCGGAGGCTCACTGTG
p53	CGGCTACCACATCCAAGGAA	GCTGGAATTACCGCGGCT
GAPDH	CCTCAACTACATGGTCTACA	CCTGGAAGATGGTGATGG

**Table 4:** Proteins in the specific band observed by His pull-down assay were identified by mass spectrometry, and the results are shown in Figure 2.

Accession	Description	MW [kDa]	Sum PEP Score
I3LUP6	Nucleophosmin	32.6	29.389
F2Z594	High mobility group protein B1	25.3	28.364
A0A4X1TPT6	Annexin A2	38.7	8.369

## Data Availability

The data presented in this study are not publicly available but are available upon reasonable request.
